# Rhodamine 101 Conjugates of Triterpenoic Amides Are of Comparable Cytotoxicity as Their Rhodamine B Analogs

**DOI:** 10.3390/molecules27072220

**Published:** 2022-03-29

**Authors:** Niels V. Heise, Daniel Major, Sophie Hoenke, Marie Kozubek, Immo Serbian, René Csuk

**Affiliations:** Department of Organic Chemistry, Martin-Luther University Halle-Wittenberg, Kurt-Mothes Str. 2, D-06120 Halle (Saale), Germany; niels.heise@chemie.uni-halle.de (N.V.H.); daniel.major@student.uni-halle.de (D.M.); sophie.hoenke@chemie.uni-halle.de (S.H.); marie.kozubek@chemie.uni-halle.de (M.K.); immoserbian@gmail.com (I.S.)

**Keywords:** triterpenoic acid, ursolic acid, oleanolic acid, betulinic acid, rhodamine B, rhodamine 101, cytotoxicity

## Abstract

Pentacyclic triterpenoic acids (betulinic, oleanolic, ursolic, and platanic acid) were selected and subjected to acetylation followed by the formation of amides derived from either piperazine or homopiperazine. These amides were coupled with either rhodamine B or rhodamine 101. All of these compounds were screened for their cytotoxic activity in SRB assays. As a result, the cytotoxicity of the parent acids was low but increased slightly upon their acetylation while a significant increase in cytotoxicity was observed for piperazinyl and homopiperazinyl amides. A tremendous improvement in cytotoxicity was observed; however, for the rhodamine B and rhodamine 101 conjugates, and compound **27**, an ursolic acid derived homopiperazinyl amide holding a rhodamine 101 residue showed an EC_50_ = 0.05 µM for A2780 ovarian cancer cells while being less cytotoxic for non-malignant fibroblasts. To date, the rhodamine 101 derivatives presented here are the first examples of triterpene derivatives holding a rhodamine residue different from rhodamine B.

## 1. Introduction

Despite significant progress, cancer therapy still falls short of the expectations placed in it many years ago [1,2]. The prognosis for a complete cure is very good for some types of cancer, but still poor for many others, especially when regular screening is taken into account. The high cost of therapy [3,4,5] is often offset especially for cancers that are difficult to treat by only a slight increase in life expectancy and, at the same time, a significantly reduced quality of life. Thus, the survival rate [6] for testicular cancer is approximately 98% while for pancreatic cancer it is about 1%. The main reason for the reduced quality of life and, thus, a reduced compliance by the affected patients is more or less often insufficient selectivity of the chemotherapeutic agents used. As a consequence, there has been no lack of attempts to improve the efficacy but also to reduce the side effects caused by antitumor drugs (such as weight loss, hair loss, etc.). Furthermore, many different strategies have been tested for a successful drug targeting of tumors—whereby the real problem is usually not the solid primary tumor but the metastases that have already formed and spread throughout the body. These attempts [7] included the use of micelles [8], antibodies [9], liposomes [10], polymers but also of drug-loaded nanoparticles [7].

Although first described several years ago, so-called mitocans (i.e., mitochondria targeting anticancer drugs) [11,12,13,14,15,16] are currently experiencing a scientific renaissance. Mitocans, which specifically induce a programmed cell death in tumor cells, can be considered as one of the most innovative therapeutic approaches of drug targeting of the “next generation”. In the past, we could already show with some examples that compounds derived from pentacyclic triterpenes (such as ursolic, oleanolic, betulinic, platanic, glycyrrhetinic, β-boswellic, tormentic, euspaphic, or asiatic acid) exhibit high cytotoxicity, and can act as mitocans [17,18,19,20,21,22]. These mitocans might cause either membrane permeabilization but also the opening of the mitochondrial permeability pore [16,17]. However, the deactivation of mitochondrial enzymes cannot completely be ruled out [17].

**Figure 1:**
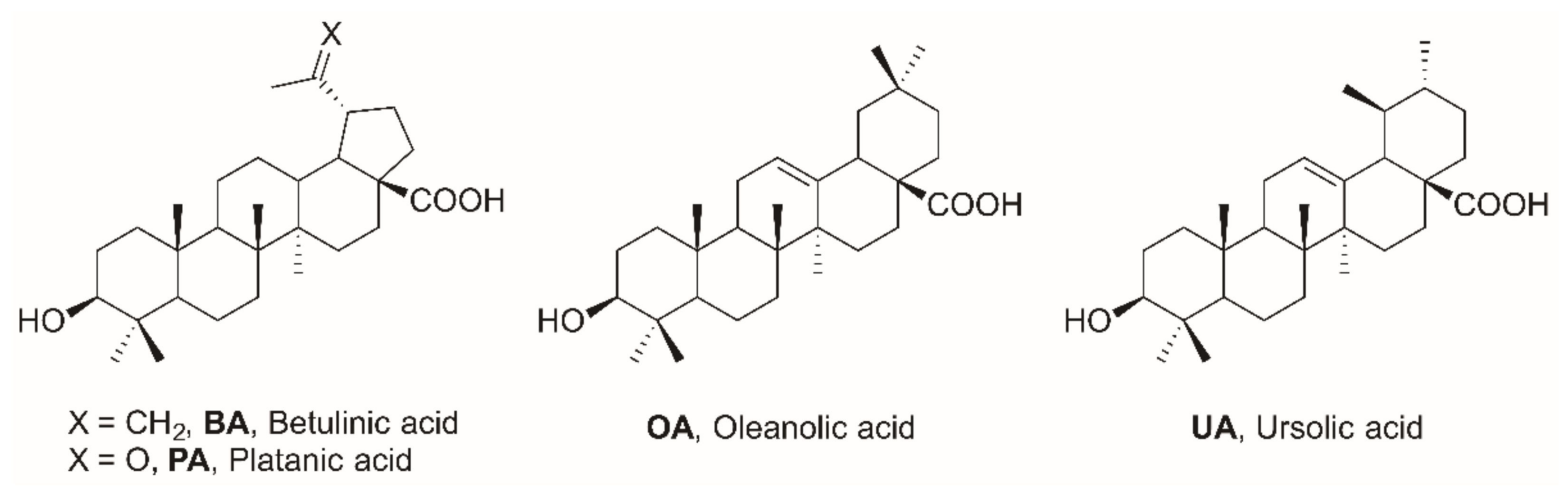
Structure of betulinic acid (**BA**), platanic acid (**PA**), oleanolic acid (**OA**), and **UA** (ursolic acid).

This cytotoxicity (but also to some extent their pronounced tumor/non-tumor cell selectivity) seems to depend on many parameters. On the one hand, this concerns a dependence on the type of terpene (Figure 1) used: compounds derived from dehydroabietylamine [23] were—by and large—less cytotoxic than those with a pentacyclic triterpenoid backbone [17]. Amides at position C-28 were mostly more active than the analogous esters [22], whereas a direct attachment of a rhodamine B moiety to the triterpenoid backbone resulted in compounds of significantly lowered selectivity [17]. Therefore, the use of a suitable spacer is of crucial importance. Furthermore, triterpene/rhodamine B hybrids holding an ethylenediamine spacer [20] were significantly less active than those with a piperazine spacer; in some cases, the use of a homopiperazine spacer [17] proved successful. However, the presence of a distal cationic center alone is not sufficient for achieving good cytotoxic activity [17,24,25,26]. Only special delocalized lipophilic cations are useful for a successful mitochondria-targeted chemotherapy. Thereby, quaternary ammonium salts [24] but also malachite green-derived compounds [27] proved to be significantly less cytotoxic than their rhodamine B analogs [17]. Furthermore, the presence of a rhodamine residue is of crucial importance, which is why we decided to extend our studies to rhodamine B and other rhodamines, and to investigate especially the synthesis and cytotoxic activity of (homo)-piperazinyl-spaced triterpenes holding a rhodamine 101 residue in more detail, and to compare their cytotoxic activity with those carrying a rhodamine B unit.

## 2. Results

**Scheme 1:**
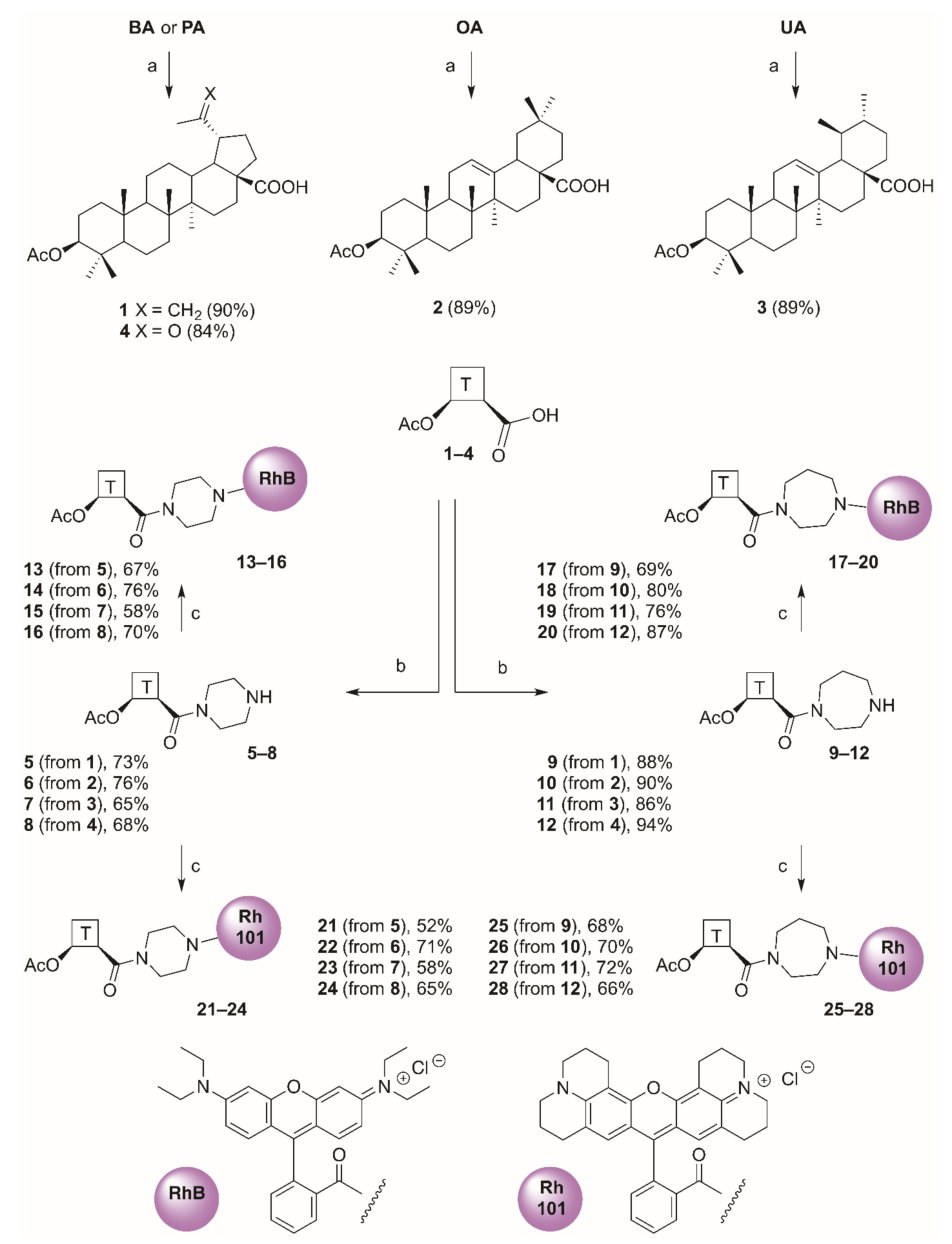
Reactions and conditions: (**a**) Ac_2_O, NEt_3_, DMAP (cat.), DCM, 20 °C, 1 d; (**b**) (COCl)_2_, DMF (cat.), DCM, then DCM (homo)piperazine, 20 °C, 1 h; (**c**) rhodamine B or rhodamine 101, (COCl)_2_, NEt_3_, DMAP (cat.), DCM, 20 °C, 1 d; T—triterpenoic acid; OA—oleanolic acid; UA—ursolic acid; BA—betulinic acid; PA—platanic acid; Rh B—rhodamine B; Rh 101—rhodamine 101.

Acetylation (Scheme 1) of betulinic (**BA**, Figure 1), oleanolic (**OA**), ursolic (**UA**), and platanic (**PA**) acid gave well known acetates **1**–**4**; their carboxyl group was activated with oxalyl chloride followed by the addition of either piperazine or homopiperazine to furnish amides **5**–**8** and **9**–**12**, respectively. Activation of rhodamine B or rhodamine 101 with oxalyl chloride and reaction with amides **5**–**12** furnished piperazine/rhodamine B derived conjugates **13**–**16** and **17**–**20** as well as rhodamine 101 derived hybrids **21**–**24** and **25**–**28**, respectively. All of these conjugates were violet in color, hence, indicating the presence of an intact cationic rhodamine moiety. This is regarded as a prerequisite for obtaining high cytotoxicity due to interaction with the mitochondrial membrane(s). 

The cytotoxicity of the compounds was determined in sulforhodamine B (SRB) assays employing several human tumor cell lines (A375, HT29, MCF-7, A2780, FaDu) as well as two non-malignant cell lines (NIH 3T3, HEK293). The results from these assays are summarized in Table 1, Table 2, Table 3 and Table 4.

Table 1 shows the results from the SRB assays for the parent compounds and their acetates. Except for **BA** and **UA**, all other triterpenoids held EC_50_ values > 30 µM (cut-off of the assay) for the cancer cell lines but also for the non-malignant fibroblasts NIH 3T3. Acetates **1**–**4** showed slightly improved cytotoxicity (except **PA** derived **4**); by-and-large, EC_50_ values between 7.2 µM (**1** for FaDu cells) and 21.3 (**1** for A375 cells) were observed. Highest cytotoxicity was found for **1** and FaDu cells, for **2** with respect to A2780 and for **3** also with A2780 cells, respectively. Interestingly, **PA** derived acetate **4** was not cytotoxic at all within the limits of the assay.

Significant improvement was observed for the piperazinyl amides (Table 2), and EC_50_ values between 1.00 (**5** for HT29) and 3.86 (for **PA** derived **8** and HT29 cells) were determined. Except for the latter, all EC_50_ values were smaller than 3 µM. While **5** was cytotoxic with an EC_50_ = 1.5 µM for A375 cells, its homopiperazinyl analog **9** was significantly less active (EC_50_ = 18.7 µM). However, by-and-large, the cytotoxicity of the homopiperazinyl derivatives **9**–**12** was of the same order as that of the piperazinyl analogs **5**–**8**.

A dramatic improvement of cytotoxicity, however, was observed for the piperazinyl and homopiperazinyl spacered triterpenoic acid–rhodamine B conjugates **13–20** (Table 3).

Thereby, all of the compounds showed high cytotoxicity for all human tumor cell lines; EC_50_ values ranged from EC_50_ = 0.02 µM (compound **18** and A2780 cells) to EC_50_ = 0.76 µM (compound **17** and A375 cells). Thus, the former compound was as cytotoxic as standard doxorubicin.

Previously, we have shown the high cytotoxicity of several rhodamine B conjugates. Hence, it became of interest to investigate whether this high cytotoxicity is limited to rhodamine B conjugates or can also be found in conjugates holding a rhodamine 101 scaffold. As a result (Table 4), conjugates holding either a piperazinyl or homopiperazinyl spacer were only slightly less cytotoxic than those holding a rhodamine B moiety.

**Figure 2:**
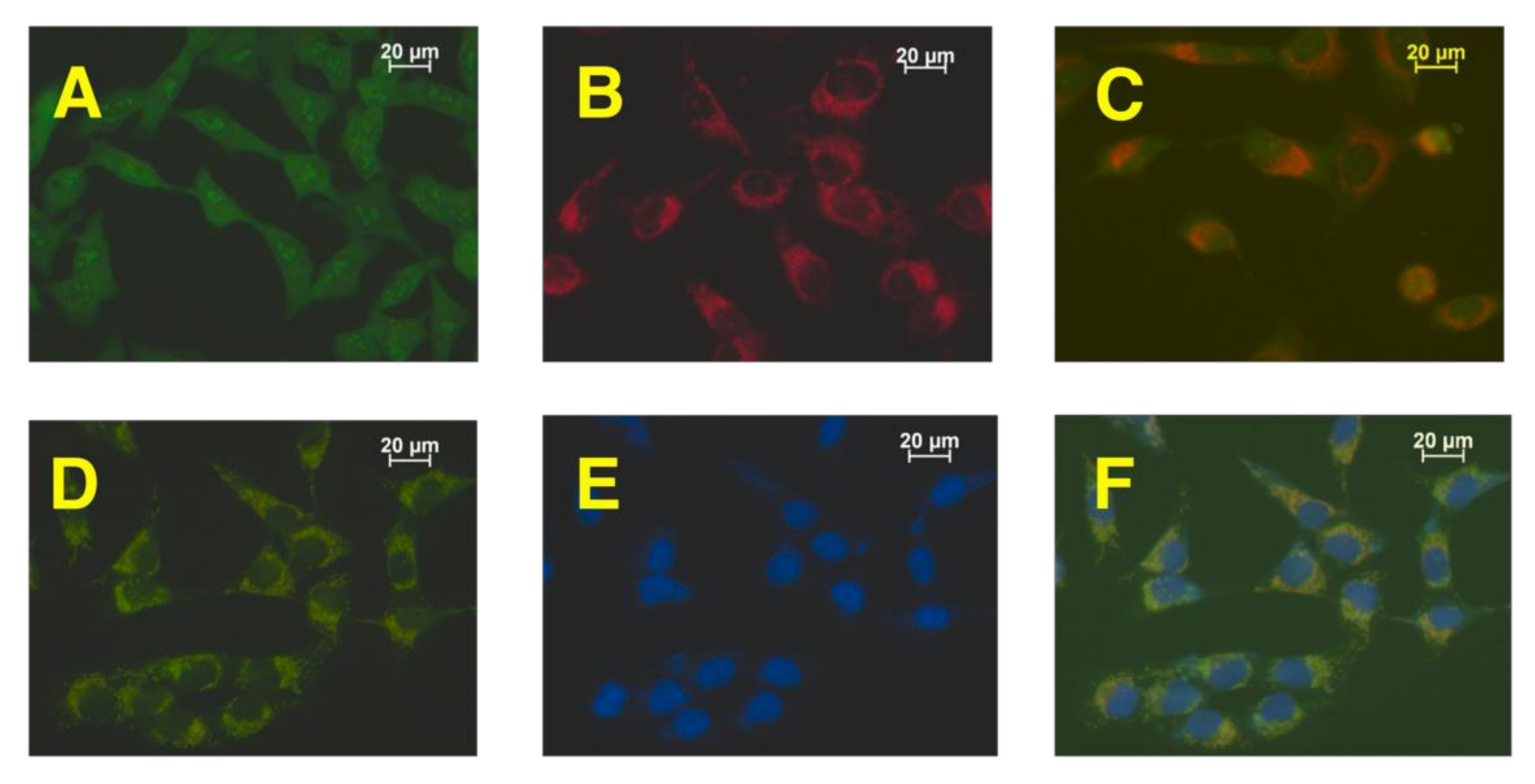
Staining experiments: A375 cells, 24 h; (**A**) control (AO); (**B**) in the presence of **26**; (**C**) merged (AO, **26**); (**D**) control (AO); (**E**) Hoechst 33,342 staining; (**F**) merged (Hoechst 33,342).

Interestingly enough, in this series of compounds, **UA** derived **27** (carrying a homopiperazine spacer and a rhodamine 101 residue) held the highest cytotoxicity, and the EC_50_ values for this compound were as low as EC_50_ = 0.05 µM (A2780 cells). Cytotoxicity for non-malignant fibroblasts NIH 3T3 were approximately five times lower for both rhodamine scaffolds. Extra staining experiments of A375 cells (acridine orange (AO), Hoechst 33342, rhodamine 123 (Figure 2)) showed **26** to act as a mitocan.

**Figure 3:**
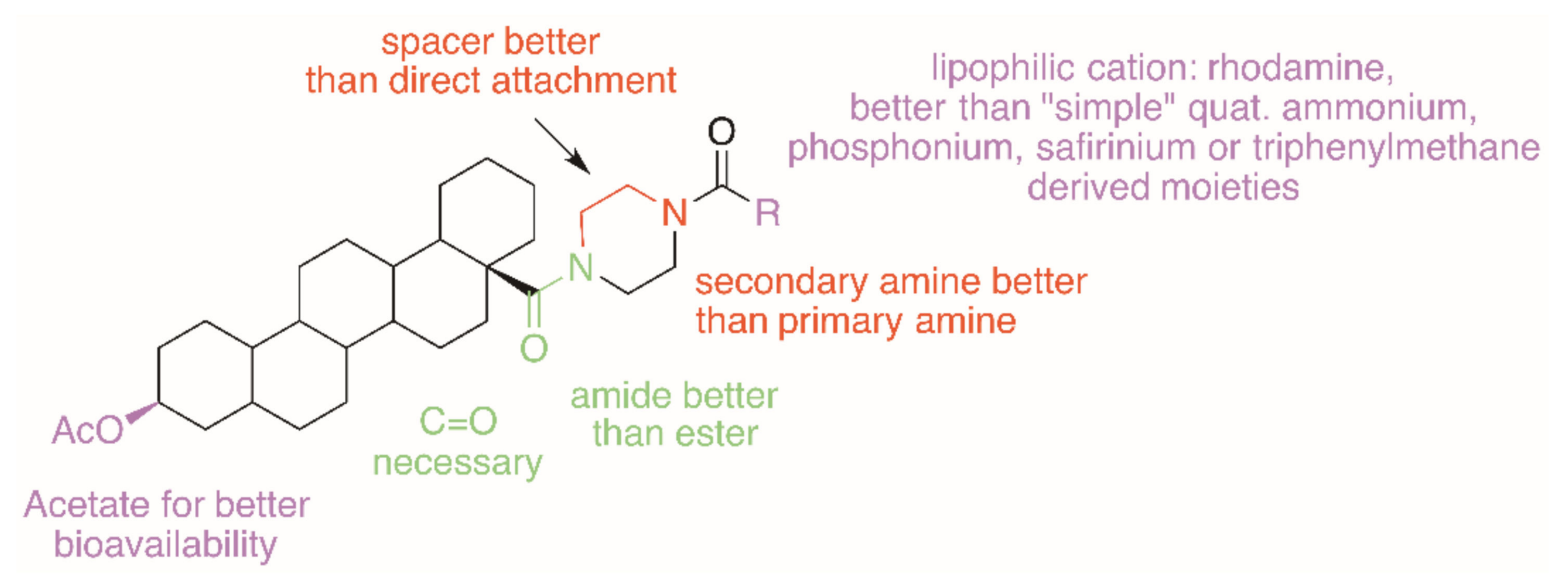
Hitherto known SAR parameters for obtaining pentacyclic triterpenoids of high cytotoxicity.

A summary of the hitherto known structural prerequisites to obtain pentacyclic triterpenoids of high cytotoxicity is depicted in Figure 3.

## 3. Conclusions

Four representative pentacyclic triterpenoic acids (**BA**, **OA**, **UA,** and **PA**) were selected for a systematic evaluation of cytotoxic derivatives. As a result—and as exemplified for A2780 cancer cells—the cytotoxicity of the parent acids is low but increased slightly upon their acetylation. A significant increase in cytotoxicity was observed when acetates **1–4** were transformed into their piperazinyl amides **5**–**8**. For the latter, compounds EC_50_ values between EC_50_ = 2.6 to 1.7 µM have been determined. The same trend was observed for the homopiperazinyl derivatives **9**–**12**. Interestingly, betulinic acid derived **9** (EC_50_ = 12.0 µM) was significantly less cytotoxic than its piperazinyl derivative **5** (EC_50_ = 1.9 µM). A tremendous improvement in cytotoxicity was observed, however, for the rhodamine conjugates, and EC_50_ values between EC_50_ = 0.05–0.032 µM were observed for the piperazinyl rhodamine B conjugates. The corresponding piperazinyl rhodamine 101 conjugates were of comparable bioactivity (EC_50_ = 0.09–0.17 µM). A similar trend was observed for the homopiperazinyl rhodamine conjugates, and EC_50_ = 0.02–0.45 µM (for rhodamine B derived **17**–**20**) and EC_50_ = 0.05–0.19 µM (for rhodamine 101 derived **25**–**28**) were determined. Thus, it can be concluded that an optimal combination of pentacyclic triterpene, a suitable spacer and a lipophilic cationic residue must be found to achieve good cytotoxic activity. It was shown that both piperazinyl and homopiperazinyl spacers are equally suitable to serve as anchors for the binding of either rhodamine B or rhodamine 101. Furthermore, the conjugates derived from either rhodamine B or rhodamine 101 are of comparable cytotoxicity.

## 4. Experimental

NMR spectra were recorded using the Varian spectrometers (Darmstadt, Germany) DD2 and VNMRS (400 and 500 MHz, respectively). MS spectra were taken on a Advion expression^L^ CMS mass spectrometer (Ithaca, NY, USA); positive ion polarity mode, solvent: methanol, solvent flow: 0.2 mL/min, spray voltage: 5.17 kV, source voltage: 77 V, APCI corona discharge: 4.2 μA, capillary temperature: 250 °C, capillary voltage: 180 V, sheath gas: N_2_). Thin-layer chromatography was performed on pre-coated silica gel plates supplied by Macherey-Nagel (Düren, Germany). IR spectra were recorded on a Spectrum 1000 FT-IR-spectrometer from Perkin Elmer (Rodgau, Germany). The UV/Vis-spectra were recorded on a Lambda 14 spectrometer from Perkin Elmer (Rodgau, Germany); optical rotations were measured at 20 °C using a JASCO-P2000 instrument (JASCO Germany GmbH, Pfungstadt, Germany) The melting points were determined using the Leica hot stage microscope Galen III (Leica Biosystems, Nussloch, Germany) and are uncorrected. The solvents were dried according to usual procedures. Microanalyses were performed with an Elementar Vario EL (CHNS) instrument (Elementar Analysensysteme GmbH, Elementar-Straße 1, D-63505, Langenselbold, Germany).

### 4.1. General Procedure for the Synthesis of Acetates ***1***–***4*** (GPA)

To a solution of the triterpenoic acid (**OA**, **UA**, **BA**, **PA**, 1 equiv.) in dry DCM, acetic anhydride (3 equiv.), triethylamine (3 equiv.), and DMAP (cat.) were added, and the mixture was stirred at 20 °C for 1 day. Usual aqueous work-up followed by re-crystallization from ethanol furnished products **1**–**4**.

### 4.2. 3β-Acetyloxy-lup-20(29)-en-28-oic Acid *(**1**)*

Following GPA, compound **1** (4.90 g, 90%) was obtained from **BA** as a colorless solid; R_f_ = 0.59 (n-hexane/ethyl acetate, 4:1); m.p. 277–279 °C (lit.: [28] 277–278 °C); [α]_D_ = +20.7° (*c* 0.42, CHCl_3_) [(lit.: [29] [α]_D_ = +20.7° (*c* 0.42, CHCl_3_)]; MS (ESI, MeOH): *m*/*z* 487.1 (32%, [M − H]^−^, 995.0 (100%, [2M − H]^−^, 1018.6 (29% [2M − 2H + Na]^−^).

### 4.3. 3β-Acetyloxy-olean-12-en-oic Acid *(**2**)*

Following GPA, compound **2** (2.45 g, 89%) was obtained from **OA** as a colorless solid; R_f_ = 0.72 (toluene/ethyl acetate/heptane/formic acid, 80:26:10:5); m.p. 286–289 °C (lit.: [30] 264–265 °C); [α]_D_ = +69.4° (*c* 0.30, CHCl_3_) [(lit.: [31] [α]_D_ = +74.0° (*c* 1.0, CHCl_3_)]; MS (ESI, MeOH): *m*/*z* 499.4 ([100%, M + H]^+^), 516.3 (36%, [M + NH_4_]^+^, 521.5 [35%, [M + Na]^+^.

### 4.4. 3β-Acetyloxy-urs-12-en-oic Acid *(**3**)*

Following GPA, compound **3** (2.41 g, 89%) was obtained from **UA** as a colorless solid; R_f_ = 0.57 (n-hexane/ethyl acetate, 3:1); m. p. 281–283 °C (lit.: [32] 280 °C); [α]_D_ = +66.5° (*c* 0.42, CHCl_3_) [lit.: [33] [α]_D_ = +63.5° (*c* 0.5, CHCl_3_)]; MS (ESI, MeOH): *m*/*z* 499.3 (17%, [M + H]^+^), 521.2 (31%, [M + H]^+^), 1019.4 (100%, [2M + Na]^+^).

### 4.5. 3β-Acetyloxy-20-oxo-30-norlupan-28-oic Acid *(**4**)*

Following GPA, compound **4** (6.9 g, 84%) was obtained from **PA** as a colorless solid; R_f_ = 0.50 (toluene/ethyl acetate/heptane/formic acid, 80:26:10.5); m.p. 265–268 °C (lit.: [34] 252–255 °C); [α]_D_ = −9.4° (*c* 0.32, CHCl_3_) [(lit.: [34] [α]_D_ = −9.5° (*c* 0.5, CHCl_3_)]; MS (ESI, MeOH): *m*/*z* 999.5 (100%, [2M − H]^−^).

### 4.6. General Procedure for the Synthesis of Acetylated (homo)piperazinyl Amides ***5***–***12*** (GPB)

To a solution of the acetylated triterpenoic acid **1**–**4** (1 equiv.) in dry DCM, DMF (cat.) and oxalyl chloride (4 equiv.) were added followed by the addition of (homo)piperazine (4 equiv.). After stirring for 1h at 20 °C followed by usual aqueous work-up and column chromatography, products **5**–**12** were obtained.

### 4.7. 3β-Acetyloxy-28-(1-piperazinyl)-lup-20(29)en-28-one *(**5**)*

Following GPB from **1** (2.5 g, 5 mmol) and piperazine (1.6 g, 20.0 mmol), compound **5** (2.07 g, 73%) was obtained as a colorless solid; R_f_ = 0.40 (SiO_2_, CHCl_3_/MeOH, 9:1); m.p. = 166–173 °C (lit.: [35] 162–167 °C); [α]_D_ = −1.4° (*c* 0.21, MeOH), [lit.: [35] [α]_D_ = −1.8° (*c* 0.32, MeOH); MS (ESI, MeOH): *m*/*z* (%) = 567.3 ([100%, M + H]^+^).

### 4.8. 3β-Acetyloxy-28-(1-piperazinyl)-olean-12-en-28-one *(**6**)*

Following GPB from **2** (2.5 g, 5.0 mmol) and piperazine (1.6 g, 29 mmol), **6** (2.16 g, 76%) was obtained as a colorless solid; R_f_ = 0.40 (SiO_2_, CHCl_3_/MeOH, 9:1); m.p. = 172–175 °C (lit.: [35] 173–175 °C); [α]_D_ = +23.4° (*c* 0.18, CHCl_3_), [lit.: [35] [α]_D_ = +26.6° (*c* 0.35, MeOH); MS (ESI, MeOH): *m*/*z* (%) = 567.4 (100%, [M + H]^+^).

### 4.9. 3β-Acetyloxy-28-(1-piperazinyl)-urs-12-en-28-one *(**7**)*

Following GPB from **3** (2.5 g, 5.0 mmol) and piperazine (1.6 g, 20.0 mmol), **7** (1.84 g, 65%) was obtained as a colorless solid; R_f_ = 0.40 (SiO_2_, CHCl_3_/MeOH, 9:1); m.p. = 187–190 °C (lit.: [35] 187–188 °C); [α]_D_ = +25.1° (*c* 0.24, MeOH), [lit.: [35] [α]_D_ = +24.5° (*c* 0.29, MeOH); MS (ESI, MeOH): *m*/*z* (%) = 567.4 (100%, [M + H]^+^).

### 4.10. 3β-Acetyloxy-28-(1-piperazinyl)-30-norlupane-20,28-dione *(**8**)*

Following GPB from **4** (2.5 g, 5.0 mmol) and piperazine (1.6 g, 20.0 mmol), **17** (1.93 g, 68%) was obtained as a colorless solid; R_f_ = 0.40 (SiO_2_, CHCl_3_/MeOH, 9:1); m.p. = 127–130 °C (lit.: [20] 115–125 °C); [α]_D_ = −20.3° (*c* 0.13, CHCl_3_); MS (ESI, MeOH): *m*/*z* (%) = 569.3 (100%, [M + H]^+^).

### 4.11. 3β-Acetyloxy-28-(1-homopiperazinyl)-lup-20(29)en-28-one *(**9**)*

Following GPB from **1** (1.0 g, 2.02 mmol) and homopiperazine (9.8 g, 8.0 mmol), **9** (1.02 g, 88%) was obtained as a colorless solid; R_f_ = 0.4 (SiO_2_, CHCl_3_/MeOH, 9:1); m.p. 190–193°C (lit.: [21] 196–199 °C); [α]_D_ = +107.6° (*c* 0.20, CHCl_3_), [lit.: [21] [α]_D_ = +109.8° (*c* 0.38, CHCl_3_); MS (ESI, MeOH): *m*/*z* = 581.4 (100%, [M + H]^+^).

### 4.12. 3β-Acetyloxy-28-(1-homopiperazinyl)-olean-12-en-28-one *(**10**)*

Following GPB from **2** (1.0 g, 2.02 mmol) and homopiperazine (0.8 g, 8.0 mmol), **10** (1.4 g, 90%) was obtained as a colorless solid; R_f_ = 0.4 (SiO_2_, CHCl_3_/MeOH, 9:1); m.p. 182–185 °C (lit.: [21] 187–190 °C); [α]_D_ = 12.4° (*c* 0.14, CHCl_3_), [lit.: [21] [α]_D_ = +9.9° (*c* 0.35, CHCl_3_); MS (ESI, MeOH): *m*/*z* = 581.4 (100%, [M + H]^+^).

### 4.13. 3β-Acetyloxy28-(1-homopierazinyl)-urs-12-en-28-one *(**11**)*

Following GPB from **3** (1.0 g, 2.02 mmol) and homopiperazine (0.8 g, 8.0 mmol), **11** (1.1 g, 86%) was obtained as a colorless solid; R_f_ = 0.4 (SiO_2_, CHCl_3_/MeOH, 9:1); m.p. 171–175 °C (lit.: [21] 178–180 °C); [α]_D_ = +27.0° (*c* 0.21, CHCl_3_), [lit.: [21] [α]_D_ = +29.7° (*c* 0.34, CHCl_3_); MS (ESI, MeOH): *m*/*z* = 581.4 (100%, [M + H]^+^, 100%).

### 4.14. 3β-Acetyloxy-28-(1-homopiperazinyl)-30-norlupane-20,28-dione *(**12**)*

Following GPB from **4** (1.0 g, 2.02 mmol) and homopiperazine (0.8 g, 8.0 mmol), **12** (1.1 g, 94%) was obtained as a colorless solid; R_f_ = 0.4 (SiO_2_, CHCl_3_/MeOH, 9:1); m.p. 162–165 °C; [α]_D_ = −29.2° (*c* 0.16, CHCl_3_); IR (ATR): ν = 2941m, 2866w, 1732m, 1708m, 1622m, 1464m, 1453m, 1408m, 1367m, 1244vs, 1191m, 1149m, 1133m, 1109w, 1026m, 979m, 947w, 934w, 900w, 750s, 665m, 610w, 557m, 506m, 455w, 410w cm^−1^; ^1^H NMR (400 MHz, CDCl_3_): δ = 4.44 (dd, *J* = 10.5, 5.5 Hz, 1H, 3-H), 3.89–3.60 (m, 4H, 33-H), 3.40–3.09 (m, 5H, 19-H, 33-H), 2.62 (td, *J* = 12.1, 4.1 Hz, 1H, 13-H), 2.25 (s, 2H, 35-H), 2.17 (s, 3H, 29-H), 2.14–2.06 (m, 2H, 16-H_a_ + 18-H), 2.02 (s, 3H, 32-H), 1.97–1.84 (m, 2H, 21-H_a_ + 22-H_a_), 1.68–1.14 (m, 15H + 1-H_a_ + 2-H + 6-H_a_ + 6-H_b_ + 7-H + 9-H + 11-H_a_ + 11-H_b_ + 15-H + 16-H_b_ + 21-H_b_ + 22-H_b_), 1.06–0.98 (m, 2H, 12-H), 0.96 (s, 4H, 1-H_b_ + 27-H), 0.89 (s, 3H, 26-H), 0.82 (s, 3H, 24-H), 0.82 (s, 3H, 25-H), 0.81 (s, 3H, 23-H), 0.79–0.74 (m, 1H, 5-H) ppm; ^13^C NMR (101 MHz, CDCl_3_): δ = 213.0 (C-20), 175.0 (C-28), 171.2 (C-31), 81.0 (C-3), 55.4 (C-5), 54.9 (C-17), 52.6 (C-18), 50.6 (C-9), 49.9 (C-19), 46.6 (C-33 + C-34), 41.8 (C-14), 40.6 (C-8), 38.3 (C-1), 37.8 (C-4), 37.1 (C-10), 36.0 (C-13), 35.7 (C-22), 34.1 (C-7), 31.7 (C-16), 30.3 (C-29), 29.9 (C-15), 28.6 (C-21), 27.9 (C-24), 27.3 (C-12), 25.7 (C-35), 23.6 (C-2), 21.3 (C-32), 21.1 (C-11), 18.1 (C-6), 16.4 (C-23), 16.2 (C-25), 15.9 (C-26), 14.7 (C-27) ppm; MS (ESI, MeOH): *m*/*z* = 583.3 (100%, [M + H]^+^; analysis calcd for C_36_H_58_N_2_O_4_ (582.87): C 74.18, H 10.03, N 4.81; found: C 73.95, H 10.34, N 4.63.

### 4.15. General Procedure for the Synthesis of the Rhodamine Conjugates ***13***–***28*** (GPC)

To a solution of the respective rhodamine (1.15 eq.) in dry DCM at 0 °C, DMF (cat.) and oxalyl chloride (4 eq.) were added (vide supra). This acid chloride was slowly added to the solution of the respective triterpene (1 eq. in DCM) in the presence of triethylamine (1 eq.). After stirring for 1 day at 20 °C, aqueous work-up was carried out as usual and the residue was purified by column chromatography.

### 4.16. 9-[2-[[4-(3β-Acetyloxy-28-oxo-lup-20(29)en-28-yl)-1-piperazinyl]carbonyl]phenyl]-3,6-bis(diethylamino)-xanthylium chloride *(**13**)*

Following GPC from **5** (180 mg, 0.32 mmol) and rhodamine B, **13** (220 mg, 67%) was obtained as a pink solid; R_f_ = 0.39 (SiO_2_, CHCl_3_/MeOH, 9:1); m.p. 247–252 °C (lit.: [20] m.p. 246–250 °C); MS (ESI, MeOH): *m*/*z* = 991.6 (100%, [M − Cl]^+^).

### 4.17. 9-[2-[[4-(3β-Acetyloxy-28-oxo-olean-12-en-28-yl)-1-piperazinyl]carbonyl]phenyl]-3,6-bis(diethylamino)-xanthylium chloride *(**14**)*

Following GPC from **6** (180 mg, 0.32 mmol) and rhodamine B, **14** (250 mg, 76%) was obtained as a pink solid; R_f_ = 0.40 (SiO_2_, CHCl_3_/MeOH, 9:1); m.p. 246–252 °C (lit.: [20] 245–248 °C); MS (ESI, MeOH): *m*/*z* = 991.9 (100%, [M − Cl]^+^).

### 4.18. 9-[2-[[4-(3β-Acetyloxy-28-oxo-ursan-12-en-28-yl)-1-piperazinyl]carbonyl]phenyl]-3,6-bis(diethylamino)-xanthylium chloride *(**15**)*

Following GPC from **7** (180 mg, 0.32 mmol) and rhodamine B, **15** (190 mg, 58%) was obtained as a pink solid; R_f_ = 0.40 (SiO_2_, CHCl_3_/MeOH, 9:1); m.p. 245–251 °C (lit.: [20] 243–245 °C); MS (ESI, MeOH): *m*/*z* = 991.7 (100%, [M − Cl]^+^).

### 4.19. 9-[2-[[4-(3β-Acetyloxy-20,28-dioxo-30-norlupan-28-yl)-1-piperazinyl]carbonyl]phenyl]-3,6-bis(diethylamino)-xanthylium chloride *(**16**)*

Following GPC from **8** (180 mg, 0.32 mmol) and rhodamine B, **16** (230 mg, 70%) was obtained as a pink solid; R_f_ = 0.37 (SiO_2_, CHCl_3_/MeOH, 9:1); m.p. 247–254 °C (lit.: [20] 235–243 °C); MS (ESI, MeOH): *m*/*z* = 993.7 (100%, [M − Cl]^+^).

### 4.20. 9-[2-[[4-(3β-Acetyloxy-28-oxo-30-norlupan-28-yl)-1-homopiperazinyl]carbonyl]phenyl]- 3,6-bis(diethylamino)-xanthylium chloride *(**17**)*

Following GPC from **9** (260 mg, 0.32 mmol) and rhodamine B, **17** (229 mg, 69%) was obtained as a pink solid; R_f_ = 0.50 (SiO_2_, MeCN/CH_2_Cl_2_/H_2_O, 10:1:1); m.p. 261–266 °C (lit.: [21] 256–260 °C); MS (ESI, MeOH): *m*/*z* = 1005.7 (100%, [M − Cl]^+^).

### 4.21. 9-[2-[[4-(3β-Acetyloxy-28-oxo-olean-12-en-28-yl]-1-homopiperazinyl]carbonyl]phenyl]- 3,6-bis(diethylamino)-xanthylium chloride *(**18**)*

Following GPC from **10** (270 mg, 0.32 mmol) and rhodamine B, **18** (267 mg, 80%) was obtained as a pink solid; R_f_ = 0.52 (SiO_2_, MeCN/CH_2_Cl_2_/H_2_O, 10:1:1); m.p. 235–241 °C (lit.: [21] 238–245 °C); MS (ESI, MeOH): *m*/*z* = 1005.8 (100%, [M − Cl]^+^).

### 4.22. 9-[2-[[4-(3β-Acetyloxy-28-oxo-urs-12-en-28-yl]-1-homopiperazinyl]carbonyl]phenyl]- 3,6-bis(diethylamino)-xanthylium chloride *(**19**)*

Following GPC from **11** (270 mg, 0.32 mmol) and rhodamine B, **19** (253 mg, 76%) was obtained as a pink solid; R_f_ = 0.51 (SiO_2_, MeCN/CH_2_Cl_2_/H_2_O, 10:1:1); m.p. 238–246 °C (lit.: [21] 238–245 °C); MS (ESI, MeOH): *m*/*z* = 1005.7 (100%, [M − Cl]^+^).

### 4.23. 9-[2-[[4-(3β-Acetyloxy-20,28-dioxo-30-norlupan-28-yl)-1-homopiperazinyl]carbonyl]phenyl]-3,6-bis(diethylamino)-xanthylium chloride *(**20**)*

Following GPC from **10** (125 mg, 0.21 mmol) and rhodamine B, **18** (190 mg, 87%); was obtained as a pink solid; m.p. 248–250 °C (decomp.); R_f_ = 0.30 (SiO_2_, CHCl_3_/MeOH, 9:1); UV-Vis (CHCl_3_): λ_max_ (log ε) = 562 nm (5.05); IR (ATR): ν = 2937w, 1730w, 1585s, 1466m, 1411m, 1334s, 1273m, 1244m, 1178s, 1131m, 1072m, 977w, 920w, 822w, 746m, 683m cm^−1^; ^1^H NMR (500 MHz, CDCl_3_): δ = 7.66–7.57 (*m*, 2H, 42-H, 43-H), 7.42–7.36 (*m*, 1H, 40-H), 7.31–7.27 (*m*, 1H, 41-H), 7.25–7.15 (*m*, 2H, 50-H), 6.99–6.83 (*m*, 2H, 49-H), 6.79–6.68 (*m*, 2H, 47-H), 4.40 (*dd*, *J* = 10.4, 5.6 Hz, 1H, 3-H), 3.85–3.31 (*m*, 16H, 32-H, 33-H, 34-H, 36-H, 51-H), 3.27–3.12 (*m*, 1H, 13-H), 2.72–2.57 (*m*, 1H, 19-H), 2.11 (*s*, 3H, 29-H), 2.18–2.06 (*m*, 1H, 16-H_a_), 1.98 (*s*, 3H, 31-H), 1.96 (*s,* 1H, 18-H), 1.95–0.97 (*m*, 21H, 22-H_a_, 21-H_a_, 35-H, 1-H_a_, 2-H, 16-H_b_, 21-H_b_, 6-H, 22-H_b_, 11-H_a_, 7-H, 11-H_b_, 9-H, 15-H, 12-H), 1.32–1.26 (*m*, 12H, 52-H), 0.92 (*s*, 3H, 27-H), 0.89 (*s*, 3H, 24-H), 0.94–0.86 (*m*, 1H, 1-H_b_), 0.79 (*s*, 9H, 23-H, 25-H, 26-H), 0.73 (*s*, 1H, 5-H_b_) ppm; ^13^C NMR (126 MHz, CDCl_3_): δ = 212.8 (C-20), 174.7 (C-28), 170.7 (30 C-), 168.3 (C-37), 157.5 (C-48), 157.5 (C-44), 155.4 C- (46), 136.5 (C-38) 135.9 (C-39), 132.1 (C-50), 130.1 (C-41), 129.6 (C-42), 129.4 (C-43), 126.3 (C-40), 113.9 (C-49), 113.4 (C-45), 96.2 (C-47), 80.7 (C-3), 55.3 (C-5), 54.7 (C-17), 52.7 (C-18), 50.4 (C-9), 50.0 (C-19), 46.0 (C-51), 41.6 (C-14), 40.4 (C-8), 38.2 (C-1), 37.6 (C-4), 36.9 (C-10), 35.8 (C-13), 35.3 (C-22), 34.1 (C-7), 31.7 (C-35), 31.2 (C-16), 30.0 (C-29), 29.7 (C-15), 28.7 (C-21), 27.7 (C-23), 27.2 (C-12), 23.5 (C-2), 21.1 (C-31), 21.0 (C-11), 18.0 (C-6), 16.3 (C-25), 16.1 (C-26), 16.0 (C-24), 14.4 (C-27), 12.5 (C-52). ppm; MS (ESI, MeOH): *m*/*z* 1007 (100%, [M]^+^); analysis calculated for C_64_H_85_ClN_4_O_6_ (1041.84): C 73.78, H 8.22, N 5.38; found: C 73.55, H 8.41, N 5.19.

### 4.24. 3β-Acetyloxy-28-[4-[3-(2,3,6,7,12,13,16,17-octahydro-1H,5H,11H,15H-pyrido[3,2,1-ij] pyrido[1”,2”,3”:1′,8′]quinolino[6′,5′:5,6]pyrano[2,3-f]quinolin-4-ium-9-yl)benzoyl]piperazine-1-yl]-28-oxo-lup-20(29)-en chloride *(**21**)*

Following GPC from **5** (0.25 g, 0.44 mmol) and rhodamine 101 (0.25 g, 0.51 mmol), **21** (0.24 g, 52%) was obtained as a pink colored solid; R_f_ = 0.4 (SiO_2_, CHCl_3_/MeOH, 9:1); m.p. >300 °C; IR (ATR): ν = 3400w, 2939w, 2862w, 1728w, 1630m, 1595m, 1542w, 1493m, 1434m, 1360m, 1294vs, 1267s, 1248s, 1195s, 1182s, 1099s, 1035m, 1003m, 978m, 896w, 827w, 772m, 747m, 637m, 560m, 506m, 421s cm^−1^; ^1^H NMR (400 MHz, CDCl_3_): δ = 7.67 (dd, *J* = 5.7, 3.3 Hz, 2H, 37-H, 39-H), 7.53–7.48 (m, 1H, 38-H), 7.31 (dd, *J* = 5.7, 3.2 Hz, 1H, 40-H), 6.68 (d, *J* = 6.0 Hz, 2H, 48-H), 4.68 (s, 1H, 29-H_a_), 4.56 (s, 1H, 29-H_b_), 4.44 (dd, *J* = 10.2, 5.9 Hz, 1H, 3-H), 3.60–3.48 (m, 4H, 49-H), 3.48–3.40 (m, 4H, 52-H), 3.40–3.31 (m, 8H, 33-H + 34-H), 3.04–2.97 (m, 4H, 54-H), 2.91 (dt, *J* = 11.9, 6.1 Hz, 1H, 19-H), 2.81–2.74 (m, 1H, 13-H), 2.74–2.60 (m, 4H, 51-H), 2.14–2.04 (m, 4H, 53-H), 2.02 (s, 3H, 32-H), 1.98–1.91 (m, 4H, 50-H), 1.77 (q, *J* = 33.5, 10.4 Hz, 4H, 12-H + 16-H_a_ + 22-H_a_), 1.65 (s, 3H, 30-H), 1.63–1.44 (m, 8H, 1-H + 2-H + 6-H_a_ + 11-H + 16-H_b_ + 18-H), 1.44–1.08 (m, 10H, 6-H_b_ + 7-H + 9-H + 15-H + 21-H + 22-H_b_), 0.92 (s, 3H, 27-H), 0.88 (s, 3H, 26-H), 0.82 (s, 6H, 23-H + 24-H), 0.81 (s, 3H, 25-H), 0.77 (s, 1H, 5-H) ppm; ^13^C NMR (101 MHz, CDCl_3_): δ = 174.1 (C-28), 171.0 (C-31), 167.9 (C-35), 162.7 (C-20), 152.0 (C-43), 151.2 (C-44), 150.9 (C-46), 134.8 (C-41), 131.8 (C-36), 130.7 (C-40), 130.2 (C-39), 129.7 (C-37), 127.5 (C-38), 126.5 (C-48), 123.6 (C-45), 123.6 (C-45), 113.2 (C-42), 109.4 (C-29), 105.4 (C-47), 80.9 (C-3), 55.5 (C-5), 54.6 (C-17), 52.5 (C-18), 51.0 (C-49), 50.7 (C-9), 50.5 (C-52,), 45.7 (C-19), 41.9 (C-14), 40.6 (C-8), 38.4 (C-1), 37.8 (C-4), 37.1 (C-10), 36.9 (C-13), 35.8 (C-22), 34.3 (C-7), 32.5 (C-16), 31.2 (C-21), 29.8 (C-15), 27.9 (C-24), 27.7 (C-51), 25.5 (C-12), 23.7 (C-2), 21.3 (C-32), 20.6 (C-11, C-50), 19.9 (C-54), 19.6 (C-53), 19.5 (C-30), 18.1 (C-6), 16.5 (C-23), 16.2 (C-25), 16.1 (C-26), 14.6 (C-27) ppm; MS (ESI, MeOH): *m*/*z* = 1039.3 (100%, [M − Cl]^+^); analysis calculated for C_68_H_87_N_4_O_5_Cl (1075.92): C 75.91, H 8.15, N 5.21; found: C 75.77, H 8.36, N 5.05.

### 4.25. 3β-Acetyloxy-28-[4-[3-(2,3,6,7,12,13,16,17-octahydro-1H,5H,11H,15H-pyrido[3,2,1-ij]pyrido[1”,2”,3”:1′,8′]quinolino[6′,5′:5,6]pyrano[2,3-f]quinolin-4-ium-9-yl)benzoyl]piperazine-1-yl]-28-oxoolean-12-en chloride *(**22**)*

Following GPC from **6** (0.25 g, 0.44 mmol) and rhodamine 101 (0.25 g, 0.51 mmol), **22** (0.33 g, 71%) was obtained as a pink solid; R_f_ = 0.4 (SiO_2_, CHCl_3_/MeOH, 9:1); m.p. >300 °C; IR (ATR): ν = 3398w, 2941w, 2859w, 1728w, 1631m, 1594s, 1543w, 1494s, 1458m, 1435m, 1361m, 1295Vs, 1267s, 1248s, 1196s, 1182s, 1100s, 1077m, 1035m, 1002m, 897w, 862w, 773w, 733m, 640m, 560m, 498m, 421s cm^−1^; ^1^H NMR (500 MHz, CDCl_3_) δ = 7.70–7.66 (m, 2H, 37-H, 39-H), 7.53–7.49 (m, 1H, 38-H), 7.34–7.30 (m, 1H, 40-H), 6.67 (d, *J* = 7.5 Hz, 2H, 48-H), 5.23 (t, *J* = 3.5 Hz, 1H, 12-H), 4.48 (dd, *J* = 9.9, 6.0 Hz, 1H, 3-H), 3.63–3.51 (m, 4H, 49-H), 3.52–3.40 (m, 4H, 52-H), 3.39–3.29 (m, 8H, 33-H + 34-H), 3.09–3 (m, 4H, 54-H), 3.00–2.96 (m, 1H, 18-H), 2.76–2.62 (m, 4H, 51-H), 2.15–2.07 (m, 5H, 16-H_a_ + 53-H), 2.04 (s, 3H, 32-H), 2.01–1.91 (m, 4H, 50-H), 1.89–1.81 (m, 1H, 11-H), 1.71–1.48 (m, 10H, 1-H_a_ + 2-H + 6-H_a_ + 7-H + 9-H + 15-H_a_ + 16-H_b_ + 19-H_a_), 1.48–1.13 (m, 7H, 6-H_b_ + 19-H_b_ + 21-H + 22-H_a_ + 22-H_b_), 1.11 (s, 3H, 27-H), 1.08–0.94 (m, 2H, 1-H_b_ + 15-H_b_), 0.91 (s, 3H, 25-H), 0.89 (s, 3H, 29-H), 0.88 (s, 3H, 30-H), 0.86 (s, 3H, 24-H), 0.85 (s, 3H, 23-H), 0.82 (s, 1H, 5-H), 0.66 (s, 3H, 26-H) ppm; ^13^C NMR (126 MHz, CDCl_3_): δ = 176.4 (C-28), 171.0 (C-31), 167.8 (C-35), 152.8 (C-43), 152.0 (C-44), 152.0 (C-44), 151.3 (C-46), 151.2 (C-46), 144.4 (C-13), 134.8 (C-41), 131.8 (C-36), 130.8 (C-40), 130.2 (C-39), 129.7 (C-37), 127.4 (C-36), 126.5 (C-48), 126.5 (C-48), 123.6 (C-45), 123.5 (C-45), 121.7 (C-12), 113.3 (C-42), 105.5 (C-47), 105.5 (C-47), 80.8 (C-3), 55.3 (C-5), 51.0 (C-49, 49), 50.6 (C-52), 47.6 (C-9, 33, 34), 47.5 (C-17), 46.2 (C-19), 43.5 (C-18), 41.8 (C-14), 39.1 (C-8), 38.0 (C-1), 37.7 (C-4), 37.0 (C-10), 33.9 (C-21), 32.9 (C-30), 32.8 (C-22), 30.3 (C-20), 30.0 (C-7), 28.0 (C-24), 27.8 (C-15), 27.7 (C-51), 25.9 (C-27), 24.0 (C-29), 23.5 (C-2), 23.3 (C-11), 22.5 (C-16), 21.3 (C-32), 20.6 (C-50), 19.9 (C-54), 19.7 (C-53), 18.2 (C-6), 16.9 (C-26), 16.7 (C-23), 15.4 (C-25) ppm; MS (ESI, MeOH): *m*/*z* = 1039.3 (100%, [M − Cl]^+^); analysis calculated for C_68_H_87_N_4_O_5_Cl (1075.64): C 75.91, H 8.15, N 5.21; found: C 75.72, H 8.29, N 5.01.

### 4.26. 3β-Acetyloxy-28-[4-[3-(2,3,6,7,12,13,16,17-octahydro-1H,5H,11H,15H-pyrido[3,2,1-ij] pyrido[1”,2”,3”:1′,8′]quinolino[6′,5′:5,6]pyrano[2,3-f]quinolin-4-ium-9-yl)benzoyl]piperazine-1-yl]-28-oxo-urs-12-en chloride *(**23**)*

Following GPC from **7** (0.25 g, 0.44 mmol) and rhodamine 101 (0.25 g, 0.51 mmol), **23** (0.27 g, 58%) was obtained as a pink solid; R_f_ = 0.4 (SiO_2_, CHCl_3_/MeOH, 9:1); m.p. >300 °C; IR (ATR): ν = 3392w, 2926w, 2867w, 1728w, 1626m, 1594s, 1542w, 1493s, 1457m, 1435m, 1361m, 1294vs, 1266s, 1246s, 1196s, 1181s, 1099s, 1035m, 1004m, 897w, 863w, 773m, 732m, 653m, 561m, 420s cm^−1^; ^1^H NMR (500 MHz, CDCl_3_): δ = 7.71–7.65 (m, 2H, 37-H, 39-H), 7.54–7.48 (m, 1H, 38-H), 7.34–7.30 (m, 1H, 40-H), 6.67 (d, *J* = 4.7 Hz, 2H, 48-H), 5.17 (s, 1H, 12-H), 4.48 (dd, *J* = 10.4, 5.5 Hz, 1H, 3-H), 3.56 (dt, *J* = 17.2, 6.1 Hz, 4H, 49-H), 3.51–3.41 (m, 4H, 52-H), 3.33 (s, 8H, 33-H + 34-H), 3.02 (q, *J* = 6.0 Hz, 4H, 54-H), 2.68 (ddt, *J* = 22.7, 15.6, 7.3 Hz, 4H, 51-H), 2.39–2.32 (m, 1H, 18-H), 2.15–2.06 (m, 4H, 53-H), 2.04 (s, 3H, 32-H), 1.99–1.94 (m, 4H, 50-H), 1.91–1.87 (m, 1H + 11-H_a_), 1.79–1.57 (m, 7H + 1-H_a_ + 2-H + 11-H_b_ + 16-H + 22-H_a_), 1.56–1.40 (m, 5H, 6-H_a_ + 7-H_a_ + 9-H + 21-H_a_ + 22-H_b_), 1.40–1.20 (m, 4H, 6-H_b_ + 7-H_b_ + 19-H + 21-H_b_), 1.05 (s, 6H, 1-H_b_ + 15-H + 27-H), 0.99–0.95 (m, 1H, 20-H), 0.93 (s, 3H, 30-H), 0.91 (s, 3H, 25-H), 0.86 (s, 3H, 24-H), 0.86 (s, 3H, 26-H), 0.84 (s, 3H, 29-H), 0.81–0.80 (m, 1H, 5-H), 0.67 (s, 3H, 23-H) ppm; ^13^C NMR (126 MHz, CDCl_3_): δ = 174.8 (C-28), 171.0 (C-31), 167.8 (C-35), 152.8 (C-46), 152.0 (C-43), 151.3 (C-44), 151.2 (C-44), 134.8 (C-41), 130.8 (C-40), 130.2 (C-37), 129.8 (C-39), 127.5 (C-38), 126.5 (C-48), 125.2 (C-12), 123.6, 123.5 (C-45), 113.2 (C-42), 105.5 (C-47), 105.5 (C-47), 80.8 (C-3), 55.3 (C-5, 18), 51.0 (C-49), 50.6 (C-52), 48.6 (C-17), 47.5 (C-9, C-33, C-34), 42.1 (C-14), 39.8 (C-19), 39.4 (C-8), 38.7 (C-20), 34.4 (C-22), 32.9 (C-7), 30.4 (C-21), 28.1 (C-15), 28.1 (C-24), 27.7 (C-51), 27.7 (C-51), 23.5 (C-2, 16, 27), 23.3 (C-11), 21.3 (C-30), 21.2 (C-32), 20.6 (C-50), 19.9 (C-54), 19.7 (C-53), 18.2 (C-6), 17.4 (C-29), 16.9 (C-26), 16.7 (C-23), 15.5 (C-25) ppm; MS (ESI, MeOH): *m*/*z* = 1039.4 ([M − Cl]^+^, 100%); analysis calculated for C_68_H_87_N_4_O_5_Cl (1075.64): C 75.91, H 8.15, N 5.21; found: C 75.64, H 8.29, N 4.96.

### 4.27. 3β-Acetyloxy-28-[4-[3-(2,3,6,7,12,13,16,17-octahydro-1H,5H,11H,15H-pyrido[3,2,1-ij]pyrido[1”,2”,3”:1′,8′]quinolino[6′,5′:5,6]pyrano[2,3-f]quinolin-4-ium-9-yl)benzoyl]piperazine-1-yl]-20,28-dioxo-30-norlupan-12-en chloride *(**24**)*

Following GPC from **8** (0.25 g, 0.44 mmol) and rhodamine 101 (0.25 g, 0.51 mmol), **24** (0.30 g, 65%) was obtained as a pink solid; R_f_ = 0.4 (SiO_2_, CHCl_3_/MeOH, 9:1); m.p. >300 °C; IR (ATR): ν = 3396w, 2940w, 2863w, 1727w, 1627m, 1594s, 1543w, 1493s, 1459m, 1446m, 1439m, 1419m, 1361m, 1295Vs, 1267s, 1195s, 1182s, 1099s, 1035m, 1004m, 979m, 897w, 863w, 773m, 732m, 561m, 505m, 420s cm^−1^; ^1^H NMR (500 MHz, CDCl_3_): δ = 7.70–7.67 (m, 2H, 37-H, 39-H), 7.53–7.49 (m, 1H, 38-H), 7.33–7.30 (m, 1H, 40-H), 6.66 (d, *J* = 6.8 Hz, 2H, 48-H), 4.44 (dd, *J* = 10.9, 5.2 Hz, 1H, 3-H), 3.62–3.51 (m, 4H, 49-H), 3.51–3.43 (m, 4H, 52-H), 3.43–3.29 (m, 8H, 33-H + 34-H), 3.15 (td, *J* = 11.1, 3.1 Hz, 1H, 19-H), 3.06–2.98 (m, 4H, 54-H), 2.78–2.63 (m, 4H, 51-H), 2.58 (td, *J* = 12.1, 3.8 Hz, 1H, 13-H), 2.14 (s, 3H, 29-H), 2.13–2.08 (m, 4H, 53-H), 2.08–2.04 (m, 1H, 18-H), 2.02 (s, 3H, 32-H), 1.97 (q, *J* = 6.9 Hz, 5H, 16-H_a_ + 50-H), 1.88–1.80 (m, 2H, 21-H_a_ + 22-H_a_), 1.67–1.54 (m, 4H, 1-H_a_ + 2-H + 16-H_b_), 1.54–0.98 (m, 14H, 6-H_a_ + 6-H_b_ + 7-H + 9-H + 11-H_a_ + 11-H_b_ + 12-H + 15-H + 21-H_b_ + 22-H_b_), 0.95 (s, 4H, 1-H_b_ + 27-H), 0.87 (s, 3H, 26-H), 0.82 (s, 3H, 24-H), 0.82 (s, 3H, 25-H), 0.81 (s, 3H, 23-H), 0.79–0.75 (m, 1H, 5-H) ppm; ^13^C NMR (126 MHz, CDCl_3_) δ = 212.6 (C-20), 173.9 (C-28), 170.9 (C-31), 167.8 (C-35), 152.8 (C-43), 152.0 (C-44), 152.0 (C-44), 151.3 (C-46), 151.2 (C-46), 134.7 (C-41), 131.9 (C-36), 130.8 (C-40), 130.3 (C-39), 129.7 (C-37), 127.4 (C-38), 126.5 (C-48), 123.6 (C-45), 123.5 (C-45), 113.2 (C-42), 105.5 (C-47), 55.4 (C-5), 54.5 (C-17), 52.5 (C-18), 51.0 (C-49), 51.0 (C-49), 50.6 (C-52), 50.6 (C-52), 50.5 (C-9), 50.0 (C-19), 41.7 (C-14), 40.5 (C-8), 38.3 (C-1), 37.8 (C-4), 37.1 (C-10), 35.9 (C-13), 35.6 (C-22), 34.2 (C-7), 32.0 (C-16), 30.2 (C-29), 29.8 (C-15), 28.7 (C-21), 27.9 (C-24), 27.7 (C-51), 27.4 (C-12), 23.6 (C-2), 21.3 (C-32), 21.1 (C-11), 20.6 (C-50), 19.9 (C-54), 19.7 (C-53), 18.1 (C-6), 16.5 (C-23), 16.2 (C-25), 16.0 (C-26), 14.6 (C-27) ppm; MS (ESI, MeOH): *m*/*z* = 1041.3 ([M − Cl]^+^, 100%); analysis calculated for C_67_H_85_N_4_O_6_Cl (1077.89): C 74.66, H 7.95, N 5.20; found: C 74.50, H 8.14, N 5.03.

### 4.28. 3β-Acetyloxy-28-[4-[3-(2,3,6,7,12,13,16,17-octahydro-1H,5H,11H,15H-pyrido[3,2,1-ij] pyrido[1”,2”,3”:1′,8′]quinolino[6′,5′:5,6]pyrano[2,3-f]quinolin-4-ium-9-yl)benzoyl]homopiperazine-1-yl]-28-oxo-lup-20(29)-en chloride *(**25**)*

Following GPC from **9** (0.35 g, 0.60 mmol) and rhodamine 101 (0.25 g, 0.51 mmol), **25** (0.37 g, 68%) was obtained as a pink solid; R_f_ = 0.4 (SiO_2_, CHCl_3_/MeOH, 9:1); m.p. >300 °C; IR (ATR): ν = 2940w, 2865w, 1730w, 1624m, 1595s, 1542w, 1493s, 1459m, 1376m, 1361m, 1294vs, 1268s, 1247m, 1196s, 1180s, 1098s, 1035m, 1018m, 978m, 895w, 772w, 747m, 622m, 421s cm^−1^; ^1^H NMR (400 MHz, CDCl_3_): δ = 7.63–7.51 (m, 2H, 37-H, 39-H), 7.41 (t, *J* = 9.2 Hz, 1H, 38-H), 7.21 (d, *J* = 7.4 Hz, 1H, 40-H), 6.68 (dd, *J* = 26.0, 20.3 Hz, 2H, 48-H), 4.70 (d, *J* = 18.2 Hz, 1H, 29-H_a_), 4.55 (d, *J* = 17.7 Hz, 1H, 29-H_b_), 4.45–4.38 (m, 1H, 3-H), 3.85–3.62 (m, 4H, 33-H), 3.55–3.45 (m, 8H, 49-H + 52-H), 3.44–3.24 (m, 4H, 34-H), 3.08–2.91 (m, 5H, 19-H + 54-H), 2.89–2.80 (m, 1H, 13-H), 2.78–2.59 (m, 4H), 2.07 (s, 7H, 16-H_a_ + 53-H + 55-H), 1.99 (s, 3H, 32-H), 1.93 (s, 4H, 50-H), 1.83 (d, *J* = 4.6 Hz, 2H, 21-H_a_ + 22-H_a_), 1.66 (s, 2H, 12-H), 1.62 (s, 3H, 30-H), 1.55 (dd, *J* = 22.6, 11.1 Hz, 3H, 1-H + 2-H), 1.50–1.40 (m, 3H, 6-H_a_ + 16-H_b_ + 18-H), 1.40–1.30 (m, 5H, 7-H + 11-H_a_ + 21-H_b_ + 22-H_b_), 1.30–1.04 (m, 5H, 6-H_b_ + 9-H + 11-H_b_ + 15-H_a_ + 15-H_b_), 0.93–0.89 (m, 5H, 1-H + 12-H + 27-H), 0.88 (s, 3H, 26-H), 0.82–0.78 (m, 9H, 23-H + 24-H + 25-H), 0.77–0.72 (m, 1H, 5-H) ppm; ^13^C NMR (126 MHz, CDCl_3_): δ = 175.5 (C-28), 170.9 (C-31), 168.9 (C-35), 152.8, 152.0 (C-43), 151.9 (C-43), 151.3 (C-44), 151.3 (C-44), 151.2 (C-20), 151.1 (C-46), 136.0 (C-41), 131.1 (C-36), 130.5 (C-40), 129.8 (C-39), 129.5 (C-37), 127.6 (C-38), 126.6 (C-48), 126.4 (C-48), 123.4 (C-45), 123.4 (C-45), 112.9 (C-42), 109.4 (C-29), 105.4 (C-47), 105.3 (C-47), 80.9 (C-3), 55.5 (C-5), 54.8 (C-17), 52.7 (C-18), 51.0 (C-49), 50.5 (C-52), 46.1 (C-33 + 34), 45.9 (C-19), 41.9 (C-14), 40.7 (C-8), 38.4 (C-1), 37.8 (C-4), 37.1 (C-10), 36.8 (C-13), 36.0 (C-22), 34.3 (C-7), 32.3 (C-16), 31.5 (C-21), 29.9 (C-15), 28.9 (C-55), 27.9 (C-24), 27.5 (C-51), 25.5 (C-12), 23.6 (C-2), 21.3 (C-32), 21.1 (C-11), 20.6 (C-50), 19.9 (C-54), 19.7 (C-53), 19.5 (C-30), 18.2 (C-6), 16.4 (C-23), 16.2 (C-25), 16.1 (C-26), 14.6 (C-27) ppm; MS (ESI, MeOH): *m*/*z* = 1052.9 ([M − Cl]^+^, 100%); analysis calculated for C_69_H_89_N_4_O_5_Cl (1089.94): C 76.04, H 8.23, N 5.14; found: C 75.76, H 8.31, N 5.02.

### 4.29. 3β-Acetyloxy-28-[4-[3-(2,3,6,7,12,13,16,17-octahydro-1H,5H,11H,15H-pyrido[3,2,1-ij] pyrido[1′′,2′′,3′′:1′,8′]quinolino[6′,5′:5,6]pyrano[2,3-f]quinolin-4-ium-9-yl)benzoyl]homopiperazine-1-yl]-28-oxo-olean-12-en chloride *(**26**)*

Following GPC from **10** (0.25 g, 0.60 mmol) and rhodamine 101 (0.25 g, 0.51 mmol), **26** (0.38 g, 70%) was obtained as a pink solid; R_f_ = 0.4 (SiO_2_, CHCl_3_/MeOH, 9:1); m.p. > 300 °C; IR (ATR): ν = 3369vw, 2942w, 2863w, 1729w, 1623m, 1595s, 1543w, 1493s, 1459m, 1361m, 1294vs, 1268s, 1246s, 1195s, 1180s, 1150m, 1098s, 1035m, 985m, 896w, 862w, 771m, 746m, 622m, 575w, 560w, 498m, 421s cm^−1^; ^1^H NMR (400 MHz, CDCl_3_): δ = 7.63–7.58 (m, 2H, 37-H + 39-H), 7.55–7.50 (m, 1H, 38-H), 7.23–7.17 (m, 1H, 40-H), 6.72–6.60 (m, 2H, 48-H), 5.25–5.21 (m, 1H, 12-H), 4.45 (t, *J* = 7.8 Hz, 1H, 3-H), 3.95–3.60 (m, 4H, 33-H + 34-H), 3.58–3.43 (m, 8H, 49-H + 52-H), 3.41–3.11 (m, 4H, 34-H), 3.06 (s, 1H, 18-H), 3.03–2.95 (m, 4H, 54-H), 2.74–2.61 (m, 4H, 51-H), 2.14–2.02 (m, 7H, 16-H_a_ + 53-H + 55-H), 2.00 (s, 3H, 32-H), 1.94 (dq, *J* = 11.1, 5.3 Hz, 4H, 50-H), 1.88–1.79 (m, 2H + 11-H), 1.71–1.41 (m, 10H, 1-H_a_ + 2-H + 6-H_a_ + 7-H + 9-H + 15-H_a_ + 16-H_b_ + 19-H_a_), 1.41–1.26 (m, 3H, 6-H_b_, 21 + 22-H_a_), 1.26–1.12 (m, 3H, 19-H_b_ + 22-H_b_), 1.09 (s, 3H, 27-H), 1.06–0.96 (m, 2H, 1-H_b_ + 15-H_b_), 0.94 (s, 3H, 29-H), 0.88 (d, *J* = 2.0 Hz, 6H, 25-H + 30-H), 0.82 (s, 3H, 24-H), 0.81 (s, 3H, 23-H), 0.79–0.76 (m, 1H, 5-H), 0.66 (s, 3H, 26-H) ppm; ^13^C NMR (101 MHz, CDCl_3_): δ = 176.1 (C-28), 171.0 (C-31), 169.0 (C-35), 152.8 (C-43), 152.0 (C-44), 151.9 (C-44), 151.3 (C-46), 151.2 (C-46), 144.8 (C-13), 136.0 (C-41), 130.4 (C-40), 129.9 (C-39), 129.5 (C-37), 127.5 (C-36), 126.5 (C-48), 126.2 (C-48), 123.6 (C-45), 123.5 (C-45), 121.4 (C-12), 113.3 (C-42), 105.5 (C-47), 105.4 (C-47), 80.9 (C-3), 55.3, 51.0 (C-49), 50.5 (C-52), 47.7 (C-17), 47.6 (C-9), 47.6 (C-33 + 34), 46.6 (C-19), 43.5 (C-18), 42.0 (C-14), 39.0 (C-8), 38.0 (C-1), 37.7 (C-4), 37.0 (C-10), 34.0 (C-21), 32.9 (C-30), 32.7 (C-22), 30.5 (C-7), 30.3 (C-20, C-55), 28.0 (C-24), 27.8 (C-15), 27.6 (C-51), 25.9 (C-27), 24.2 (C-29), 23.5 (C-2), 23.3 (C-11), 22.5 (C-16), 21.3 (C-32), 20.6 (C-50), 19.9 (C-54), 19.7 (C-53), 19.6 (C-53), 18.2 (C-6), 17.0 (C-26), 16.6 (C-23), 15.4 (C-25) ppm; MS (ESI, MeOH): *m*/*z* = 1053.0 (100%, [M − Cl]^+^); analysis calculated for C_69_H_89_N_4_O_5_Cl (1089.94): C 76.04, H 8.23, N 5.14; found: C 75.81, H 8.40, N 4.86.

### 4.30. 3β-Acetyloxy-28-[4-[3-(2,3,6,7,12,13,16,17-octahydro-1H,5H,11H,15H-pyrido[3,2,1-ij]pyrido[1′′,2′′,3′′:1′,8′]quinolino[6′,5′:5,6]pyrano[2,3-f]quinolin-4-ium-9-yl)benzoyl]homopiperazine-1-yl]-28-oxo-urs-12-en chloride *(**27**)*

Following GPC from **11** (0.35 g, 0.60 mmol) and rhodamine 101 (0.25 g, 0.51 mmol), **27** (0.39 g, 72%) was obtained as a pink solid; R_f_ = 0.4 (SiO_2_, CHCl_3_/MeOH, 9:1); m.p. >300 °C; IR (ATR): ν = 2934w, 2866w, 1730w, 1622w, 1594w, 1543w, 1493w, 1459w, 1435w, 1376w, 1361w, 1293w, 1268w, 1246w, 1195w, 1180w, 1143w, 1098w, 1035w, 985w, 966w, 941w, 896w, 862w, 772w, 744w, 715w, 661w, 622w, 574w, 560w, 496w cm^−1^; ^1^H NMR (400 MHz, CDCl_3_): δ = 7.59 (dd, *J* = 5.7, 3.3 Hz, 2H, 37-H + 39-H), 7.43–7.34 (m, 1H, 38-H), 7.22 (dd, *J* = 5.7, 3.3 Hz, 1H, 40-H), 6.61 (d, *J* = 24.5 Hz, 2H, 48-H), 5.18–5.11 (m, 1H-H), 4.44 (dd, *J* = 11.0, 5.2 Hz, 1H, 3-H), 4.08–3.66 (m, 4H, 34-H), 3.59–3.41 (m, 8H, 49-H + 52-H), 3.38–3.07 (m, 4H, 33-H), 3.02–2.93 (m, 4H, 54-H), 2.67 (h, *J* = 9.5, 8.8 Hz, 4H, 51-H), 2.45–2.34 (m, 1H, 18-H), 2.11–2.03 (m, 4H,55-H + 53-H), 2.00 (s, 3H, 32-H), 1.96–1.81 (m, 6H, 11-H + 50-H), 1.79–1.52 (m, 7H, 1-H_a_ + 2-H + 16-H + 22-H), 1.44 (dt, *J* = 14.3, 7.8 Hz, 4H, 6-H_a_ + 7-H_a_ + 9-H + 21-H_a_), 1.37–1.11 (m, 4H, 6-H_b_ + 7-H_b_ + 19-H + 21-H_b_), 1.01 (d, *J* = 11.5 Hz, 6H, 1-H_b_ + 15-H + 27-H), 0.92 (s, 1H, 20-H), 0.88 (s, 6H, 25-H + 30-H), 0.81 (s, 6H, 24-H + 29-H), 0.80 (s, 3H, 23-H), 0.75 (s, 1H, 5-H), 0.66 (s, 3H, 26-H) ppm; ^13^C NMR (126 MHz, CDCl_3_): δ = 176.4 (C-28), 170.9 (C-31), 168.1 (C-35), 152.9 (C-46), 151.9 (C-43), 151.3 (C-44), 138.7 (C-13), 135.7 (C-41), 131.4 (C-36), 130.4 (C-40), 129.8, 129.6 (C-37), 129.5 (C-39), 127.5 (C-38), 126.8 (C-48), 125.0 (C-12), 123.7 (C-45), 113.3 (C-42), 105.3 (C-47), 105.1 (C-47), 80.9 (C-3), 55.3 (C-5, 18), 51.0 (C-49), 50.5 (C-52), 48.8 (C-17), 47.5 (C-9, 33, 34), 42.8 (C-14), 39.3 (C-8, 19), 38.7 (C-20), 38.2 (C-1), 37.6 (C-4), 36.9 (C-10), 34.4 (C-22), 32.9 (C-7), 30.4 (C-21), 30.3 (C-55), 28.0 (C-15, 24), 27.6 (C-51), 27.5 (C-51), 23.5 (C-2, 16, 27), 23.2 (C-11), 21.3 (C-30), 21.2 (C-32), 20.6 (C-50), 20.6 (C-50), 19.9 (C-54), 19.7 (C-53), 19.6 (C-53), 18.1, 17.4 (C-29), 17.0 (C-26), 16.7 (C-23), 15.5 (C-25) ppm; MS (ESI, MeOH): *m*/*z* = 1053.1 (100%, [M − Cl]^+^); analysis calculated for C_69_H_89_N_4_O_5_Cl (1089.94): C 76.04, H 8.23, N 5.14; found: C 75.76, H 8.51, N 4.97.

### 4.31. 3β-Acetyloxy-28-[4-[3-(2,3,6,7,12,13,16,17-octahydro-1H,5H,11H,15H-pyrido[3,2,1-ij]pyrido[1′′,2′′,3′′:1′,8′]quinolino[6′,5′:5,6]pyrano [2,3-f]quinolin-4-ium-9-yl)benzoyl]homopiperazine-1-yl]-20,28-dioxo-30-norlupan-12-en chloride *(**28**)*

Following GPC from **12** (0.35 g, 0.60 mmol) and rhodamine 101 (0.25 g, 0.51 mmol), **28** (0.35 g, 66%) was obtained as a pink solid; R_f_ = 0.4 (SiO_2_, CHCl_3_/MeOH, 9:1); m.p. > 300 °C; IR (ATR): ν = 3383vw, 2940w, 2863w, 1731w, 1623m, 1594s, 1542w, 1493s, 1459m, 1436m, 1376m, 1361m, 1294vs, 1268s, 1247m, 1195s, 1180s, 1143m, 1099s, 1075m, 1035m, 1018m, 978w, 897w, 862w, 746m, 623w, 561m, 506m, 421s cm^−1^; ^1^H NMR (500 MHz, CDCl_3_): δ = 7.63–7.57 (m, 2H, 37-H + 39-H), 7.41 (d, *J* = 23.5 Hz, 1H, 38-H), 7.29–7.21 (m, 1H, 40-H), 6.67 (dd, *J* = 27.4, 18.9 Hz, 2H, 48-H), 4.42 (dt, *J* = 10.6, 5.4 Hz, 1H, 3-H), 3.93–3.58 (m, 4H, 33-H), 3.58–3.40 (m, 8H, 49-H, 52-H), 3.40–3.08 (m, 5H, 19-H, 34-H), 3.06–2.91 (m, 4H, 54-H), 2.70 (d, *J* = 27.3 Hz, 5H, 13-H + 51-H), 2.12 (s, 3H, 29-H), 2.10–2.02 (m, 7H, 16-H_a_ + 53-H + 55-H), 2.00 (s, 4H, 18-H + 32-H), 1.98–1.88 (m, 5H, 22-H_a_ + 50-H), 1.87–1.80 (m, 1H, 21-H_a_), 1.66–1.52 (m, 4H, 1-H + 16-H_b_), 1.45 (q, *J* = 16.9, 12.1 Hz, 3H, 6-H_a_ + 21-H_b_ + 22-H_b_), 1.39–1.30 (m, 5H, 6-H_b_ + 7-H + 11-H_a_ + 12-H_a_), 1.29–1.19 (m, 4H, 9-H + 11-H_b_ + 15-H), 0.95–0.93 (m, 2H, 1-H + 12-H_b_), 0.91 (s, 6H, 26-H + 27-H), 0.83–0.78 (m, 9H, 23-H + 24-H + 25-H), 0.76–0.72 (m, 1H, 5-H) ppm; ^13^C NMR (126 MHz, CDCl_3_): δ = 213.0 (C-20), 174.5 (C-28), 170.9 (C-31), 168.9 (C-35), 152.6 (C-43), 151.9 (C-44), 151.3 (C-46), 136.1 (C-41), 131.1 (C-36), 130.5 (C-40), 129.8 (C-39), 129.5 (C-37), 127.5 (C-38), 126.7 (C-48), 126.2 (C-48), 123.7 (C-45), 123.5 (C-45), 112.9 (C-42), 105.3 (C-47), 80.9 (C-3), 55.5 (C-5), 54.9 (C-17), 52.8 (C-18), 51.0 (C-49), 50.6 (C-9), 50.5 (C-52), 50.2 (C-19), 48.0 (C-33 + 34), 41.8 (C-14), 40.6 (C-8), 38.4 (C-1), 37.8 (C-4), 37.1 (C-10), 36.0 (C-13), 35.6 (C-22), 34.2 (C-7), 31.9 (C-16), 30.2 (C-29), 29.9 (C-15), 29.6 (C-55), 28.9 (C-21), 27.9 (C-24), 27.6 (C-51), 27.6 (C-51), 27.3 (C-12), 23.6 (C-2), 21.3 (C-32), 21.2 (C-11), 20.6 (C-50), 19.9 (C-54), 19.7 (C-53), 19.6 (C-53), 18.2 (C-6), 16.5 (C-23), 16.2 (C-25), 16.1 (C-26), 14.7 (C-27) ppm; MS (ESI, MeOH): *m*/*z* = 1055.0 (100%, [M − Cl]^+^); analysis calcd for C_68_H_87_N_4_O_6_Cl (1091.92): C 74.80, H 8.03, N 5.13; found: C 74.61, H 8.27, N 4.95.

### 4.32. Cytotoxicity Assay (SRB)

The cell lines were obtained from Department of Oncology (Martin-Luther-University Halle Wittenberg; they were bought from ATCC; A 375 (CRL-1619), HT29 (HTB-38), MCF7 (HTM-22), A2780 (HTP-77), FaDu (HTP-43), NIH 3T3 (CRL-1658), HEK-293 (CRL-1573)). Cultures were maintained as monolayers in RPMI 1640 medium with l-glutamine (Capricorn Scientific GmbH, Ebsdorfergrund, Germany) supplemented with 10% heat inactivated fetal bovine serum (Sigma-Aldrich Chemie GmbH, Steinheim, Germany) and penicillin/streptomycin (1%, Capricorn Scientific GmbH, Ebsdorfergrund, Germany) at 37 °C in a humidified atmosphere with 5% CO_2_. The cytotoxicity of the compounds was evaluated using the sulforhodamine-B (Kiton-Red S, ABCR) micro culture colorimetric assay using confluent cells in 96-well plates with the seeding of the cells on day 0 applying appropriate cell densities to prevent confluence of the cells during the period of the experiment. On day 1, the cells were treated with six different concentrations (1, 3, 7, 12, 20, and 30 μM); thereby, the final concentration of DMSO was always <0.5%, generally regarded as non-toxic to the cells. On day 4, the supernatant medium was discarded; the cells were fixed with 10% trichloroacetic acid. After another day at 4 °C, the cells were washed in a strip washer and dyed with the SRB solution (100 μL, 0.4% in 1% acetic acid) for about 20 min to be followed by washing of the plates (four times, 1% acetic acid) and air-drying overnight. Furthermore, tris base solution (200 μL, 10 mM) was added to each well and absorbance was measured at λ = 570 nm employing a reader (96 wells, Tecan Spectra, Crailsheim, Germany). The EC_50_ values were averaged from three independent experiments performed each in triplicate calculated from semi logarithmic dose response curves applying a non-linear four-parameter Hills-slope equation (GraphPad Prism5; variables top and bottom were set to 100 and 0, respectively).

### 4.33. Acridine Orange (AO) Staining

On the first day, A375 cells were counted and seeded 1 × 10^5^ in a Petri dish (diameter 4 cm) with coverslips (22 mm × 22 mm) in 2 mL medium. After 24 h, the medium was removed, and treatment was performed with 2 mL of new medium (control) and 2 mL each of 2 times the EC_50_ concentration of compounds **27**. After 24 h, the medium was removed from the samples, the coverslip was rinsed with 1 mL of PBS (with Ca^2+^ and Mg^2+^), placed on a slide containing 20 µL of AO solution (2.5 µg/mL in PBS), and measured directly on the fluorescence microscope.

### 4.34. Hoechst 33,3342 and Rhodamine 123 Staining

On day 1, A375 cells were counted and seeded 1 × 10^5^ in a Petri dish (diameter 4 cm) with coverslips (22 mm × 22 mm) in 2 mL medium. After 24 h, the medium was removed, and treatment was performed with 1 mL of new medium containing 1 µL of compound **27** (0.08 mM solution). After another 24 h, additional treatment was performed with 1 µL rhodamine (1 mg/mL in EtOH) and 2 µL Hoechst 33342 (100 µg/mL in DMSO) for (at least) 30 min. The medium was then removed, rinsed once with PBS (with Ca^2+^ and Mg^2+^), placed on a slide containing 20 µL PBS (with Ca^2+^ and Mg^2+^), and measured directly on the fluorescence microscope.

## Figures and Tables

**Table 1:** Cytotoxicity of parent compounds **BA**, **OA**, **UA**, and **PA** as well as of their corresponding acetates **1**–**4** (EC_50_-values in µM from SRB-assays) after 72 h of treatment; the values are averaged from three independent experiments performed each in triplicate, confidence interval CI = 95%; mean ± standard mean error); n.d. not determined; doxorubicin (**DX**) was used as a positive control. Cell lines: malignant: A375 (melanoma), HT29 (colon adenocarcinoma), MCF-7 (breast adenocarcinoma), A2780 (ovarian carcinoma), FaDu (hypopharyngeal carcinoma); non-malignant: NIH 3T3 (fibroblasts).

Compound	A375	HT29	MCF-7	A2780	FaDu	NIH 3T3
**BA**	17.7 ± 0.4	14.4 ± 2.3	10.2 ± 1.2	8.8 ± 0.9	13.7 ± 0.9	16.1 ± 1.4
**OA**	>30	>30	>30	>30	>30	>30
**UA**	15.4 ± 1.0	10.6 ± 0.7	12.7 ± 0.1	11.7 ± 0.6	18.2 ± 1.7	13.1 ± 1.1
**PA**	>30	>30	>30	>30	>30	>30
**1**	19.2 ± 1.7	21.3 ± 2.0	11.0 ± 0.5	18.3 ± 0.5	7.2 ± 1.2	>30
**2**	13.0 ± 1.1	20.5 ± 1.7	12.9 ± 1.9	9.4 ± 0.5	11.8 ± 0.9	17.5 ± 1.5
**3**	11.4 ± 1.4	17.3 ± 1.5	12.1 ± 1.2	8.3 ± 0.9	10.7 ± 0.8	16.4 ± 1.7
**4**	>30	>30	>30	>30	>30	>30
**DX**	n.d.	0.9 ± 0.2	1.1 ± 0.3	0.02 ± 0.01	1.7 ± 0.3	0.06 ± 0.03

**Table 2:** Cytotoxicity of 3-*O*-acetylated triterpenoic amides **5**–**8** (piperazinyl amimides) and **9**–**12** (homopiperazinyl amides) (EC_50_-values in µM from SRB-assays) after 72 h of treatment; the values are averaged from three independent experiments performed each in triplicate, confidence interval CI = 95%; mean ± standard mean error); n.d. not determined; n.s. not soluble; doxorubicin (**DX**) was used as a positive control. Cell lines: malignant: A375 (melanoma), HT29 (colon adenocarcinoma), MCF-7 (breast adenocarcinoma), A2780 (ovarian carcinoma); non-malignant: NIH 3T3 (fibroblasts).

Compound	A375	HT29	MCF-7	A2780	NIH 3T3
**5**	1.5 ± 0.3	1.0 ± 0.1	1.4 ± 0.1	1.9 ± 0.1	0.9 ± 0.1
**6**	1.4 ± 0.2	1.3 ± 0.1	1.7 ± 0.2	1.7 ± 0.1	1.7 ± 0.1
**7**	2.0 ± 0.4	1.9 ± 0.3	1.7 ± 0.2	2.1 ± 0.1	2.1 ± 0.1
**8**	1.9 ± 0.4	3.9 ± 0.2	2.7 ± 0.3	2.6 ± 0.4	1.3 ± 0.1
**9**	18.7 ± 1.6	5.1 ± 1.1	10.7 ± 1.0	12.0 ± 0.6	18.7 ± 1.6
**10**	1.9 ± 0.9	1.9 ± 0.1	1.6 ± 0.1	2.2 ± 0.1	1.8 ± 0.2
**11**	3.2 ± 0.2	2.0 ± 0.1	2.4 ± 0.1	2.9 ± 0.24	0.9 ± 0.1
**12**	0.9 ± 0.1	2.3 ± 0.2	1.8 ± 0.2	1.6 ± 0.1	0.6 ± 0.1
**DX**	n.d.	0.9 ± 0.2	1.1 ± 0.3	0.02 ± 0.01	1.7 ± 0.3

**Table 3:** Cytotoxicity of 3-*O*-acetylated triterpenoic amides holding a distal rhodamine B unit **13**–**20** (EC_50_-values in µM from SRB-assays) after 72 h of treatment; the values are averaged from three independent experiments performed each in triplicate, confidence interval CI = 95%; mean ± standard mean error); n.d. not determined; n.s. not soluble; doxorubicin (**DX**) was used as a positive control. Cell lines: malignant: A375 (melanoma), HT29 (colon adenocarcinoma), MCF-7 (breast adenocarcinoma), A2780 (ovarian carcinoma); non-malignant: NIH 3T3 (fibroblasts).

Compound	A375	HT29	MCF-7	A2780	NIH 3T3
**13**	0.09 ± 0.01	0.151 ± 0.022	0.081 ± 0.005	0.050 ± 0.004	0.208 ± 0.034
**14**	0.09 ± 0.01	0.091 ± 0.010	0.062 ± 0.004	0.032 ± 0.001	0.137 ± 0.006
**15**	0.04 ± 0.02	0.083 ± 0.007	0.075 ± 0.005	0.038 ± 0.002	0.137 ± 0.009
**16**	0.08 ± 0.03	0.099 ± 0.016	0.070 ± 0.002	0.036 ± 0.001	0.171 ± 0.006
**17**	0.76 ± 0.09	0.28 ± 0.01	0.22 ± 0.02	0.22 ± 0.01	0.33 ± 0.07
**18**	0.04 ± 0.01	0.04 ± 0.01	0.03 ± 0.004	0.02 ± 0.003	0.14 ± 0.02
**19**	0.51 ± 0.05	0.50 ± 0.07	0.39 ± 0.04	0.45 ± 0.03	0.40 ± 0.03
**20**	0.24 ± 0.017	0.30 ± 0.027	0.15 ± 0.050	0.12 ± 0.018	0.34 ± 0.056
**DX**	n.d.	0.9 ± 0.2	1.1 ± 0.3	0.02 ± 0.01	1.7 ± 0.3

**Table 4:** Cytotoxicity of 3-*O*-acetylated triterpenoic amides holding a distal rhodamine 101 unit **13**–**20** (EC_50_-values in µM from SRB-assays) after 72 h of treatment; the values are averaged from three independent experiments performed each in triplicate, confidence interval CI = 95%; mean ± standard mean error); n.d. not determined; n.s. not soluble; doxorubicin (**DX**) was used as a positive control. Cell lines: malignant: A375 (melanoma), HT29 (colon adenocarcinoma), MCF-7 (breast adenocarcinoma), A2780 (ovarian carcinoma), HeLa (cervical cancer); non-malignant: NIH 3T3 (fibroblasts), HEK293 (human embryonic kidney).

Compound	A375	HT29	MCF7	A2780	HeLa	NIH 3T3	HEK293
**21**	0.14 ± 0.02	0.36 ± 0.06	0.21 ± 0.03	0.09 ± 0.01	0.12 ± 0.03	0.56 ± 0.07	0.05 ± 0.05
**22**	0.15 ± 0.01	0.25 ± 0.03	0.23 ± 0.02	0.11 ± 0.01	0.20 ± 0.05	0.36 ± 0.05	0.07 ± 0.02
**23**	0.16 ± 0.02	0.21 ± 0.07	0.16 ± 0.03	0.08 ± 0.02	0.12 ± 0.02	0.37 ± 0.05	0.04 ± 0.01
**24**	0.25 ± 0.04	0.26 ± 0.04	0.17 ± 0.02	0.17 ± 0.03	0.21 ± 0.02	0.26 ± 0.04	0.10 ± 0.04
**25**	0.17 ± 0.05	0.43 ± 0.08	0.22 ± 0.04	0.19 ± 0.04	0.27 ± 0.14	0.56 ± 0.07	0.12 ± 0.04
**26**	0.07 ± 0.01	0.13 ± 0.03	0.12 ± 0.03	0.07 ± 0.03	0.10 ± 0.05	0.26 ± 0.05	0.05 ± 0.01
**27**	0.06 ± 0.04	0.08 ± 0.02	0.08 ± 0.03	0.05 ± 0.02	0.09 ± 0.06	0.24 ± 0.03	0.04 ± 0.01
**28**	0.20 ± 0.03	0.35 ± 0.05	0.20 ± 0.05	0.17 ± 0.04	0.31 ± 0.15	0.39 ± 0.05	0.12 ± 0.03
**DX**	n.d.	0.9 ± 0.2	1.1 ± 0.3	0.02 ± 0.01	n.d.	1.7 ± 0.3	n.d.

## Data Availability

The data presented in this study are available on request from the corresponding author.

## Sample Availability

Samples of the compounds are available from the authors.

## References

[1] Gambardella V., Fleitas T., Tarazona N., Papaccio F., Huerta M., Rosello S., Gimeno-Valiente F., Roda D., Cervantes A. (2020). Precision Medicine to Treat Advanced Gastroesophageal Adenocarcinoma: A Work in Progress. J. Clin. Med..

[2] Gambardella V., Tarazona N., Cejalvo J.M., Lombardi P., Huerta M., Rosello S., Fleitas T., Roda D., Cervantes A. (2020). Personalized Medicine: Recent Progress in Cancer Therapy. Cancers.

[3] Hofmarcher T., Lindgren P., Wilking N., Jonsson B. (2020). The cost of cancer in Europe 2018. Eur. J. Cancer.

[4] Wilking N., Brådvik G., Lindgren P., Svedman C., Jönsson B., Hofmarcher T. (2020). A comparative study on costs of cancer and access to medicines in Europe. Ann. Oncol..

[5] Wilking N.E., Brådvik G., Lindgren P., Svedman C., Jönsson B., Hofmarcher T. (2020). A comparative study on costs of cancer and access to medicines in Europe. J. Clin. Oncol..

[6] Nuffieldtrust. https://www.nuffieldtrust.org.uk/resource/cancer-survival-rates.

[7] Lammers T., Kiessling F., Hennink W.E., Storm G. (2012). Drug targeting to tumors: Principles, pitfalls and (pre-) clinical progress. J. Control. Release.

[8] Klochkov S.G., Neganova M.E., Nikolenko V.N., Chen K., Somasundaram S.G., Kirkland C.E., Aliev G. (2021). Implications of nanotechnology for the treatment of cancer: Recent advances. Semin. Cancer Biol..

[9] Schrama D., Reisfeld R.A., Becker J.C. (2006). Antibody targeted drugs as cancer therapeutics. Nat. Rev. Drug Discov..

[10] Moosavian S.A., Bianconi V., Pirro M., Sahebkar A. (2021). Challenges and pitfalls in the development of liposomal delivery systems for cancer therapy. Semin. Cancer Biol..

[11] Chiu H.Y., Tay E.X.Y., Ong D.S.T., Taneja R. (2020). Mitochondrial Dysfunction at the Center of Cancer Therapy. Antioxid. Redox Signal..

[12] Dong L.F., Gopalan V., Holland O., Neuzil J. (2020). Mitocans Revisited: Mitochondrial Targeting as Efficient Anti-Cancer Therapy. Int. J. Mol. Sci..

[13] Fialova J.L., Raudenska M., Jakubek M., Kejik Z., Martasek P., Babula P., Matkowski A., Filipensky P., Masarik M. (2021). Novel Mitochondria-targeted Drugs for Cancer Therapy. Mini-Rev. Med. Chem..

[14] Macasoi I., Mioc A., Mioc M., Racoviceanu R., Soica I., Cheveresan A., Dehelean C., Dumitrascu V. (2020). Targeting Mitochondria through the Use of Mitocans as Emerging Anticancer Agents. Curr. Med. Chem..

[15] Mani S., Swargiary G., Singh K.K. (2020). Natural Agents Targeting Mitochondria in Cancer. Int. J. Mol. Sci..

[16] Nguyen C., Pandey S. (2019). Exploiting Mitochondrial Vulnerabilities to Trigger Apoptosis Selectively in Cancer Cells. Cancers.

[17] Hoenke S., Serbian I., Deigner H.P., Csuk R. (2020). Mitocanic Di- and Triterpenoid Rhodamine B Conjugates. Molecules.

[18] Kahnt M., Wiemann J., Fischer L., Sommerwerk S., Csuk R. (2018). Transformation of asiatic acid into a mitocanic, bimodal-acting rhodamine B conjugate of nanomolar cytotoxicity. Eur. J. Med. Chem..

[19] Serbian I., Hoenke S., Csuk R. (2020). Synthesis of some steroidal mitocans of nanomolar cytotoxicity acting by apoptosis. Eur. J. Med. Chem..

[20] Sommerwerk S., Heller L., Kerzig C., Kramell A.E., Csuk R. (2017). Rhodamine B conjugates of triterpenoic acids are cytotoxic mitocans even at nanomolar concentrations. Eur. J. Med. Chem..

[21] Wolfram R.K., Fischer L., Kluge R., Strohl D., Al-Harrasi A., Csuk R. (2018). Homopiperazine-rhodamine B adducts of triterpenoic acids are strong mitocans. Eur. J. Med. Chem..

[22] Wolfram R.K., Heller L., Csuk R. (2018). Targeting mitochondria: Esters of rhodamine B with triterpenoids are mitocanic triggers of apoptosis. Eur. J. Med. Chem..

[23] Wiemann J., Al-Harrasi A., Csuk R. (2020). Cytotoxic Dehydroabietylamine Derived Compounds. Anti-Cancer Agents Med. Chem..

[24] Brandes B., Koch L., Hoenke S., Deigner H.P., Csuk R. (2020). The presence of a cationic center is not alone decisive for the cytotoxicity of triterpene carboxylic acid amides. Steroids.

[25] Heise N., Hoenke S., Simon V., Deigner H.P., Al-Harrasi A., Csuk R. (2021). Type and position of linkage govern the cytotoxicity of oleanolic acid rhodamine B hybrids. Steroids.

[26] Hoenke S., Christoph M.A., Friedrich S., Heise N., Brandes B., Deigner H.P., Al-Harrasi A., Csuk R. (2021). The Presence of a Cyclohexyldiamine Moiety Confers Cytotoxicity to Pentacyclic Triterpenoids. Molecules.

[27] Friedrich S., Serbian I., Hoenke S., Wolfram R.K., Csuk R. (2020). Synthesis and cytotoxic evaluation of malachite green derived oleanolic and ursolic acid piperazineamides. Med. Chem. Res..

[28] Urban M., Sarek J., Klinot J., Korinkova G., Hajduch M. (2004). Synthesis of A-Seco Derivatives of Betulinic Acid with Cytotoxic Activity. J. Nat. Prod..

[29] Thibeault D., Gauthier C., Legault J., Bouchard J., Dufour P., Pichette A. (2007). Synthesis and structure-activity relationship study of cytotoxic germanicane- and lupane-type 3β-O-monodesmosidic saponins starting from betulin. Bioorg. Med. Chem..

[30] Ruzicka L., Hofmann K. (1936). Polyterpenes and polyterpenoids. C. Transpositions in the rings A and E of oleanolic acid. Carbon skeleton of pentacyclic triterpenes. Helv. Chim. Acta.

[31] Topcu G., Altiner E.N., Gozcu S., Halfon B., Aydogmus Z., Pezzuto J.M., Zhou B.-N., Kingston D.G.I. (2003). Studies on Di- and triterpenoids from Salvia staminea with cytotoxic activity. Planta Med..

[32] Corbett R.E., McDowell M.A. (1958). Extractives from the New Zealand Myrtaceae. III. Triterpene acids from the bark of Leptospermum scoparium. J. Chem. Soc..

[33] Taketa A.T.C., Breitmaier E., Schenkel E.P. (2004). Triterpenes and triterpenoidal glycosides from the fruits of Ilex paraguariensis (Mate). J. Braz. Chem. Soc..

[34] Vystrcil A., Budesinsky M. (1970). Triterpenes. XVI. Unusual epimerization of the C-19 acetyl group in 20-oxo-30-norlupane derivatives. Collect. Czech. Chem. Commun..

[35] Brandes B., Hoenke S., Fischer L., Csuk R. (2020). Design, synthesis and cytotoxicity of BODIPY FL labelled triterpenoids. Eur. J. Med. Chem..

